# Empirical Antibiotic Therapy for Ventilator-Associated Pneumonia

**DOI:** 10.3390/antibiotics2030339

**Published:** 2013-07-04

**Authors:** Joseph M. Swanson, Diana L. Wells

**Affiliations:** 1 College of Pharmacy, University of Tennessee Health Science Center, 881 Madison Ave., Suite 203, Memphis, TN 38632, USA; 2 Harrison School of Pharmacy, Auburn University, 1321 Walker Building, Auburn, AL 36849, USA; E-Mail: dlw0022@auburn.edu

**Keywords:** empirical, antibiotic, ventilator-associated pneumonia

## Abstract

Ventilator-associated pneumonia (VAP) is the most common infectious complication in the intensive care unit. It can increase duration of mechanical ventilation, length of stay, costs, and mortality. Improvements in the administration of empirical antibiotic therapy have potential to reduce the complications of VAP. This review will discuss the current data addressing empirical antibiotic therapy and the effect on mortality in patients with VAP. It will also address factors that could improve the administration of empirical antibiotics and directions for future research.

## 1. Introduction

Ventilator-associated pneumonia (VAP) is nosocomial infection that develops following at least 48 h of mechanical ventilation. It can be divided into the following two categories based on duration of mechanical ventilation: early onset VAP (occurring on days 2–4) and late onset VAP (occurring on days ≥5). A third category is based on the risk of VAP being caused by multidrug resistant pathogens, but occurring on days 2–4 [1]. Ventilator-associated pneumonia is the most common healthcare-associated infection in nearly all intensive care units (ICU) [2]. The incidence of VAP ranges from 9% to 27% and increases with the duration of mechanical ventilation [3,4,5]. It can increase duration of mechanical ventilation by 5 days, length of ICU stay by 10 days, hospitalization by 12 days, and increase hospital costs by $35,480 [6]. Interestingly, attributable mortality for VAP varies depending on the patient population. Reports range from 1% [7] to as high as 50% [8,9,10]. The lower mortality was based on a recent, more rigorous analysis [7]. Based on the high frequency and considerable morbidity, it is essential to optimize the diagnosis and management of VAP. The purpose of this review is to discuss the evidence addressing appropriate empirical antibiotic therapy and describe areas where future research should be focused to improve empirical therapy administered to patients with VAP. 

## 2. Empirical Antibiotic Therapy

### 2.1. Essential Factors

Empirical antibiotic therapy entails the initial selection of an antibiotic regimen that aims to be effective against any pathogen suspected of causing the infection. Empirical therapy has been classified as appropriate/adequate or inappropriate/inadequate based on the *in vitro* susceptibilities of the identified pathogens. Empirical regimens are deemed appropriate if the isolated pathogen(s) is sensitive (S) *in vitro* to at least one antibiotic. Regimens are deemed inappropriate if the pathogen(s) is intermediate (I) or resistant (R) *in vitro* to all of the empirical antibiotics. As inappropriate empirical antibiotic therapy (IEAT) requires a change in therapy, patients with IEAT essentially receive delayed appropriate antibiotic therapy. The duration of the delay is dependent on laboratory procedures and clinician awareness of the results. While this may seem to be an overly simplistic approach to evaluating an antibiotic regimen, appropriate empirical antibiotic therapy (AEAT) has been associated with decreased mortality in patients with many different types of infection [11,12]. A delicate balance must be maintained in selecting broad-spectrum empirical antibiotics and overuse or unnecessary use of antibiotics. This is generally addressed by a change in antibiotics (if possible) to a narrower spectrum for definitive therapy. Studies investigating the effect of AEAT on patients with VAP generally demonstrate benefits over IEAT. However, studies unable to find mortality benefits raise questions regarding the effect of AEAT/IEAT on patient outcomes. 

### 2.2. Studies Showing Benefit with AEAT

The VAP studies demonstrating an overall mortality benefit with administration of AEAT are listed in Table 1. The studies were reported from 1997 through 2007 [13,14,15,16,17,18,19,20,21]. All used quantitative cultures to confirm the diagnosis of VAP and for microbiological identification of the pathogen(s). Two studies exclusively used BAL [14,15]. The others used varying combinations of telescopic plugged catheter (TPC), protected specimen brushing (PSB), endotracheal aspirates (EA), and BAL. Two studies did not report the diagnostic threshold used in the study [16,20]. There was reasonable consensus on the thresholds reported in the studies with only minor differences in the endotracheal aspirate cutoffs of ≥10^5^ or ≥10^6^ colony forming units/milliliter (cfu/mL). The study population sizes ranged from 38 to 183 patients with a total of 1,106 patients included in the analyses. Mortality rates were reported as either crude or attributable in-hospital mortality and ranged from 8.3% to 81.6%. The considerable variability in mortality should be emphasized. Of note, the study by Clec’h *et al* was only able to find a benefit in AEAT in the less sick patients (Logistic Organ Dysfunction score of ≤4), suggesting that there are patient characteristics that may play an important role in the response to empirical antibiotic therapy and infection [18]. Viewed differently, it might be concluded that in sicker patients, there is little the clinician can do to improve their outcome. The improvement in mortality associated with AEAT is further supported by a meta-analysis published in 2008 by Kuti *et al* [22]. They included nine studies in their unadjusted analysis [13,14,15,16,17,18,23,24,25] and three studies in their adjusted analysis [15,16,26]. They found that the unadjusted odds of death when patients received IEAT was 2.34 (1.51–3.63 95% CI) times greater than with AEAT, and the adjusted odds of death when receiving IEAT was 3.03 (1.12–8.19 95% CI) times greater than with AEAT. The authors noted that there was a moderate degree of heterogeneity (*I*^2^ = 28.5%) between studies included in the unadjusted meta-analysis and a high degree of heterogeneity (*I*^2^ = 89.2%) in the adjusted analysis. They concluded that differences in the studies require the reader to interpret the results cautiously. 

### 2.3. Studies not Showing Benefit with AEAT

The VAP studies that did not demonstrate an overall mortality benefit with appropriate empirical antibiotics are included in Table 2. They were published in the years ranging from 1998 to 2012 [23,24,25,26,27,28,29]. One study exclusively used EA [26], two used only BAL [27,29], while the others used a combination of PSB, TPC, BAL and EA. The diagnostic thresholds were more consistent in these studies. However, two studies using BAL cultures had the less common threshold of ≥10^5^ cfu/mL. Some clinicians may argue that using this higher threshold would lead to antibiotic therapy being inappropriately withheld for patients who may have had a clinically significant infection. Together these studies included 801 [27,29] patients and include the largest published study (393 patients) [27] evaluating empirical therapy for VAP. Overall, the mortality rates from the studies not demonstrating benefit ranged from 3.6% to 72.7% and do not appear to be different than the studies that demonstrated a benefit with AEAT. Of note, two studies enrolled only trauma patients [27,29]. These two studies also demonstrated relatively lower mortality rates, as is usually seen in VAP studies of trauma patients. While it might be easy to dismiss these studies by concluding that VAP is not as lethal in trauma patients, the same authors have noted increased mortality in trauma patients with VAP compared to similar patients without VAP (16% versus 3% respectively, *p* = 0.0001) [30]. It is also important to clarify that while both studies in trauma patients did not find an increased mortality with one episode of IEAT, they did find an increased mortality when these trauma patients experienced greater than one episode of IEAT. While the differences in outcomes highlight potential differences in trauma patients, it also reinforces the importance of AEAT in patients with suspected VAP. 

**Table 1:** Studies showing benefit of appropriate empiric antibiotics.

Reference	ICU	Microbiological	Threshold	Number of	IEAT	AEAT	Mortality	P-value
		Confirmation	(cfu/mL)	Patients	Mortality	Mortality	Difference	
Rello *et al.*, 1997 [13]	Medical, Surgical	PSB or BAL	≥10^3^ or ≥10^4^	113	37%	15.4%	21.6%	<0.05
Luna *et al.*, 1997 [14]	Medical, Surgical	BAL	>10^4^	65	81.6%	38%	43.6%	<0.005
Kollef and Ward, 1998 [15]	Medical	Mini BAL	≥10^3^	60	56.8%	31.3%	25.5%	0.08
Iregui *et al.*, 2002 [16]	Medical	BAL or EA	N/R	107	69.7%	28.4%	41.3%	<0.01
Leroy *et al.*, 2003 [17]	N/R	PSB or BAL	≥10^3^ or ≥10^4^ or ≥10^6^	132	62%	40%	22%	0.04
		or EA						
Clec’h, 2004 [18]	Medical, Surgical	TPC or PSB	≥10^3^ or ≥10^3^ or ≥10^4^	Total: 142 LOD ≤ 4: 70	51.9% 44%	47.6% 15%	4.3% 29%	0.73 0.01
		or BAL						
Alvarez-Lerma *et al.*, 2006 [19]	General	PSB or BAL	≥10^3^ or ≥10^4^ or ≥10^5^	131	33.3%	8.6%	24.7%	0.014*
		or EA						
Teixeira *et al.*, 2007 [20]	Medical, Surgical	BAL or EA	N/R	151	50.7%	29.3%	21.4%	0.02
Garnacho-Montero *et al.*, 2007 [21]	Medical, Surgical, Trauma	PSB or BAL	>10^3^ or >10^4^ or ≥10^6^	183	72.5%	33.6%	38.9%	<0.001
		or EA						

IEAT, inappropriate empiric antibiotic therapy; AEAT, appropriate empiric antibiotic therapy; cfu, colony forming units; PSB, protected specimen brushing; BAL, bronchoalveolar lavage; EA, endotracheal aspirate; TPC, telescopic plugged catheter; N/R, not reported; LOD, logistic organ dysfunction score; * *p*-value is derived from a multivariate regression analysis.

**Table 2:** Studies showing no benefit of appropriate empiric antibiotics.

Reference	Population	Microbiological	Threshold	Number of	IEAT	AEAT	Mortality	*p*-value
		Confirmation	(cfu/mL)	Patients	Mortality	Mortality	Difference	
Sanchez-Nieto *et al.*, 1998 [24]	Trauma, Medical, Surgical	PSB or BAL	≥10^3^ or ≥10^4^ or ≥10^5^	38	43%	25%	18%	NS
		or EA						
Timsit *et al.*, 2001 [25]	Medical, Surgical	PSB or BAL	≥10^3^ or ≥10^4^	47	33%	46%	13%	0.43
Dupont, 2001 [23]	Medical, Surgical	TPC or PSB	≥10^3^ or ≥10^3^ or ≥10^4^	111	60.7%	47.3%	13.4%	0.21
		or BAL						
Fowler *et al.*, 2003 [26]	Medical, Surgical	EA	N/R	156	HR: 0.98 (0.45–2.15)			
Mueller *et al.*, 2003 [29]	Trauma	BAL	≥10^5^	82	8.8%	3.6%	5.2%	0.62
Magnotti *et al.*, 2008 [27]	Trauma	BAL	≥10^5^	393	13%	12%	1%	NS
Piskin *et al.*, 2012 [28]	General	BAL or EA	≥10^4^ or ≥10^5^	130	65.1%	72.7%	7.6%	0.497

IEAT, inappropriate empiric antibiotic therapy; AEAT, appropriate empiric antibiotic therapy; cfu, colony forming units; PSB, protected specimen brushing; BAL, bronchoalveolar lavage; EA, endotracheal aspirate; TPC, telescopic plugged catheter; N/R, not reported; HR, hazards ratio.

### 2.4. Antibiotic Timing

It has long been the belief of clinicians that beginning appropriate empirical antibiotic therapy as soon as possible is essential. In fact, the Surviving Sepsis Campaign recommends administering antibiotics within one hour of suspected severe sepsis or septic shock [31]. Interestingly, there are very few studies adequately addressing this. The best available evidence to support this approach comes from animal data in experimental sepsis. Greisman SE, DuBuy JB, and Woodward CL, investigated the effect of antibiotic timing on mortality in outbread Swiss mice [32]. They infected mice with *Escherichia coli*, *Proteus mirabilis*, or *Klebsiella pneumoniae* and evaluated the effect of aminoglycoside (either gentamicin or kanamycin) administration at varying times following inoculation. When the aminoglycoside was administered at the time of inoculation (0 h), mortality was 0%, but increased to nearly 100% as the time to antibiotic administration increased to 4–8 h following inoculation. Unfortunately, current diagnostic techniques do not immediately identify infected patients. Two studies in humans evaluated the administration of delayed appropriate empirical antibiotic therapy (DAEAT) in patients with clinically and microbiologically confirmed VAP. Unfortunately, their definitions were significantly different. Luna and colleagues defined DAEAT as appropriate therapy given within 24 h of the clinical diagnosis of VAP in patients that had a clinical pulmonary infection score (CPIS) of ≥5 on the day before the clinical diagnosis was made [33]. Because all patients had a confirmed microbiologic diagnosis based on BAL cultures, a CPIS of 5 in addition to a positive respiratory culture would be consistent with the diagnosis of VAP (*i.e.*, CPIS ≥ 7). In other words, patients were classified as receiving DAEAT because they could have been identified as having VAP at least one day sooner using the CPIS. These patients had a mean CPIS on the day before clinical diagnosis of 6.1 ± 0.2, which is just below the proposed CPIS diagnostic threshold for VAP. Patients who received DAEAT had a higher mortality rate than those with adequate therapy that was not delayed (58.3% versus 29.2% respectively, *p* = 0.007). Based on the definitions used in this study, it is difficult to determine the exact duration of delay in antibiotic therapy. If the diagnosis of VAP should have been made on the day prior to the actual diagnosis, it could be concluded that the delay in therapy was ≥24 h. Iregui *et al.* defined DAEAT as antibiotic therapy that was active *in vitro* to the identified VAP pathogen(s), and was administered ≥24 h after the diagnostic criteria for VAP were first documented [16]. Patients classified as receiving DAEAT, received this therapy an average of 28.6 ± 5.8 h following the first documentation of VAP. Those not classified as having DAEAT, received empirical therapy an average of 12.5 ± 4.2 h after identification of VAP. Crude mortality was significantly higher for patients with DAEAT (69.7% versus 28.4%, *p* < 0.01), as was VAP-attributable mortality (39.4% versus 10.8%, *p* = 0.001). Together, these two studies confirm the importance of administering AEAT within the first 24 h following VAP diagnosis. However, there are no data in humans defining the optimal time of empirical antibiotic administration. Logic would suggest that sooner is better.

### 2.5. Methods to Improve Empirical Therapy

The diagnosis of VAP is a complex subject and a minority (~40%) [34,35] of patients with clinical suspicion will actually have the infection confirmed. Additionally, major differences in diagnostic methods create question when trying to use the results of VAP studies for direct patient care. Key issues where the guidelines allow variable diagnostic methods include, quantitative versus qualitative cultures, the method of obtaining said cultures, and interpretation of the culture results [1]. Unfortunately, the differences in diagnosis are not likely to be resolved in the near future and research efforts to establish a single diagnosis are not likely to prove fruitful. Inappropriate empirical antibiotic therapy rates are alarming (~30%–70%) [14,15,16,27,29,33], and research efforts should be focused on three strategies to improve EAT: (1) improving the accuracy and timeliness of the diagnosis of VAP (2) identifying methods to ensure the initial choice of antibiotics are appropriate (3) improving the time to antibiotic administration. 

#### 2.5.1. Improving Accuracy and Timeliness of VAP Diagnosis

Methods to improve the speed at which the diagnosis is confirmed or ruled out could significantly affect the use of empirical antibiotics. The use of gram stain to confirm the diagnosis and bacterial pathogens has been evaluated with mixed results. The use of preliminary culture results has shown promise, but studies utilizing this method are from a single institution and have not been validated externally. A more detailed discussion of these two methods can be found in another review by the authors [36]. Gene expression may be an additional method to identify patients at risk of VAP or to improve the diagnosis. Martin-Loeches *et al* investigated gene expression in eight patients developing VAT or VAP [37]. They found 5,595 differentially expressed genes in the pre-infection period. Further analysis demonstrated a significant depression in expression of genes involved in the complement system signaling pathway, cyclic adenosine monophosphate pathway, and calcium signaling pathway in patients developing VAP. We measured gene expression in 20 critically ill trauma patients upon admission to the intensive care unit [38]. Patients were divided into two groups, those that developed VAP and those that did not. Between the two groups we found 810 differentially expressed genes. Of those 810, five genes (PIK3R3, ATP2A1, PI3, ADAM8, and HCN4) were found to accurately categorize 95% of patients as either VAP or no VAP using hierarchical clustering. Cobb *et al* validated preliminary data from a mouse model of VAP [39] by showing that a riboleukogram was capable of diagnosing VAP four days before the clinical diagnosis could be established [40]. These studies raise the hope that gene expression profiles will not only help to identify which patients are at greater risk of developing VAP during their stay in the ICU, but they will also provide a diagnosis of VAP before the significant clinical signs and symptoms develop. These advancements could lead to the administration of empirical antibiotics at a time much closer to the actual development of VAP. It is important to emphasize that these new technologies are not ready for clinical use. In fact, data are mixed regarding the usefulness of gene expression in predicting VAP in critically ill patients. Textoris *et al* was unable to identify biomarkers that were associated with the development of infection. They noted the transcriptional signatures of 165 trauma patients contained a generalized pattern that was more related to their trauma [41]. 

A meta-analysis conducted in 2008 compared specific antibiotic regimens for the empirical treatment of VAP [42]. The authors identified 41 randomized controlled trials that included 7,015 patients. The studies compared 29 different antibiotic regimens of commonly used broad-spectrum antibiotics. None of the specific regimens conferred a benefit in mortality. They pooled six trials to evaluate treatment failure and found meropenem to be superior to ceftazidime plus an aminoglycoside (RR 0.70, 0.53–0.93). Pooling of two trials for analysis of microbiologically confirmed VAP showed linezolid to be superior to vancomycin (RR 0.75, 0.59–0.96). Interestingly, they found no difference in mortality when comparing monotherapy to combination therapy (RR 0.94, 0.76–1.16). The results from this meta-analysis must be interpreted carefully as the authors noted the low overall quality of the included studies. Additionally, the analysis is limited by the individual antibiotic comparisons in the included studies (e.g., meropenem versus ceftazidime/aminoglycoside), as the spectrum of activity varies significantly among both monotherapy and combination therapy regimens analyzed. General conclusions from this analysis were that empirical monotherapy is not inferior to empirical combination therapy and no specific regimen was found to be superior. This makes the choice of empirical antibiotic therapy less difficult as long as bacterial susceptibilities are taken into consideration. 

#### 2.5.2. Methods to Ensure Appropriate Empirical Antibiotics

Incorporation of local antibiotic susceptibilities is essential in monitoring the effectiveness of empirical antibiotic regimens. Guidelines from the Clinical and Laboratory Standards Institute recommend establishing a unit-specific antibiogram [43]. This is extremely important as bacterial susceptibilities to antibiotics can vary by patient location [44]. The need to understand the local susceptibility patterns was recently demonstrated by Becher and colleagues. They compared a locally derived VAP empirical antibiotic algorithm to general recommendations by the guidelines [45]. Use of their algorithm for early VAP required the addition of vancomycin to ceftriaxone in order to address the 15% of early pathogens being multi-drug resistant gram-positive organisms. The addition of vancomycin improved the predicted coverage from 83% to 95% of all early pathogens. The selection of vancomycin plus piperacillin/tazobactam resulted in a predicted coverage of 93%. Our institution internally reviewed the trauma ICU-specific antibiogram and found that addition of ciprofloxacin to the standard empirical regimen of cefepime plus vancomycin would only improve the predicted coverage by 3% (94% to 97%). Such variability between institutions illustrates the necessity of utilizing local and unit-specific antibiotic susceptibilities in developing empirical antibiotic regimens for VAP. Berrazeg *et al* used MultiExperiment Viewer software and hierarchical clustering to analyze organisms included in an antibiogram [46]. The software separated pathogens by phenotypes based on antibiotic susceptibilities and geographic location. As additional pathogen information of resistant isolates was added to the software, the groupings of pathogens changed and identified new clusters for the resistant pathogens. The software is capable of revising the information in a very short time (minutes) and could be used in real time to help clinicians identify emergence of new resistant pathogens. 

#### 2.5.3. Improving the Time to Antibiotic Administration

The best method to improve the timing of AEAT is to reduce the lag time between the diagnosis of VAP and the reporting of pathogen susceptibilities. Boyer *et al* studied the use of a direct E-test on BAL fluid to determine VAP pathogen susceptibilities [47]. They compared the result of the E-test at 24 h to that of the routine laboratory diagnostic method microbroth dilution. They found agreement between the two methods to be 88.9%, and only 1.5% of disagreements would have incorrectly identified the isolate as susceptible by the E-test when it was not. The use of this technique must still be validated prospectively, but the potential would be to reduce the time from diagnosis to definitive therapy to just over 24 h. Studies aimed at a more rapid identification of pathogens and subsequent susceptibility testing are numerous [48,49,50,51,52,53]. They include techniques ranging from PCR-based identification [51,52] to real-time susceptibility testing of bacteria isolated from the initial culture [50]. Identification by PCR is rapid and can be completed within one hour. Real-time susceptibility testing does not rely on the growth of new bacterial colonies from the culture. These methods can assess the susceptibility of bacteria obtained directly from the culture. Should these methods be refined and found to be clinically useful, the concept of empirical antibiotics will become outdated and the initial antibiotics could be selected based on immediate susceptibilities. The major obstacle for applying these methods to VAP is identifying sufficient growth of pathogens to surpass the recommended diagnostic thresholds (10^3^ cfu/mL for PSB and TPC, 10^4^ or 10^5^ cfu/mL for BAL, or 10^6^ cfu/mL for EA) [1]. Many cultures contain both pathogens (growth above diagnostic threshold) and colonizing bacteria (growth below diagnostic threshold). The current tests for rapid identification are not able to quantify the bacterial load along with the susceptibility testing. It is also possible with some of these techniques that amplifiable DNA from dead bacteria (non-pathogenic) will lead to a positive result. Perhaps a combination of techniques, using preliminary cultures plus rapid susceptibility testing, could aid in more timely administration of definitive therapy. 

## 3. Conclusions

The data evaluating the effect of appropriate empirical antibiotic therapy on mortality for patients with VAP are mixed, but the majority of trials demonstrate some benefit. No specific empirical antibiotic regimen has been found to be superior, so selection should be based on local pathogen susceptibility patterns. Novel VAP diagnostic methods and pathogen susceptibility testing may enhance the clinician’s ability to improve the likelihood of administering AEAT, but further research is needed. 
